# Supplementary material for: Merkel Cell Polyomavirus Large T Antigen Induces Cellular Senescence for Host Growth Arrest and Viral Genome Persistence through Its Unique Domain
Source: Cells. 2023 Jan 20;12(3):380. doi: 10.3390/cells12030380 (PMC9913222; doi:10.3390/cells12030380)
Supplement: Supplementary file 1 [file cells-12-00380-s001.zip › SI_2023_0112_final.pdf]

Supplementary Information for

Merkel cell polyomavirus large T antigen induces cellular senescence for host growth arrest and viral genome persistence through its unique domain

Alexander M. Pham[1], Luz E. Ortiz[1], Aron E. Lukacher[1], Hyun Jin Kwun[1,2,*]

[1]Department of Microbiology and Immunology, Pennsylvania State University College of Medicine, Hershey, PA 17033, USA

[2]Penn State Cancer Institute, Hershey, PA 17033, USA

*Corresponding author: Hyun Jin Kwun
Email: hxk479@psu.edu

This PDF file includes:

Figures S1 to S11
Tables S1

Other supplementary materials for this manuscript include the following:

Supplementary Dataset S1

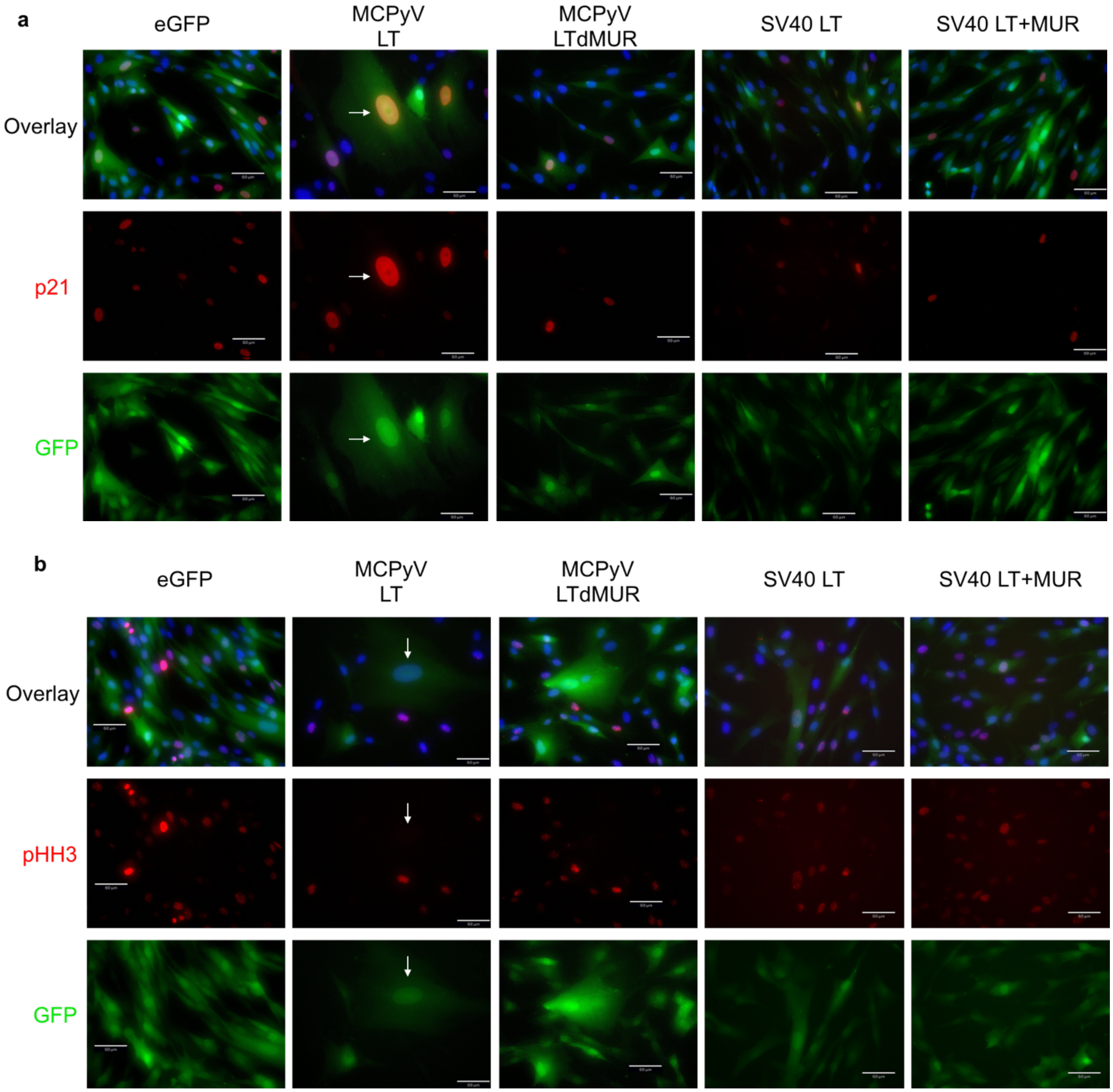

Fig. S1. MCPyV LT alters expression of growth arrest genes. Immunofluorescent analysis of p21 (red) **(a)** and pHH3 (phospho-histone H3, red) **(b)** in cells stably expressing eGFP, MCPyV LT, MCPyV LTdMUR, SV40 LT, or SV40 LT+MUR. Enlarged MCPyV LT senescent cells exhibited high p21 expression and low pHH3 levels. SV40 LT constructs did not induce p21 expression. White arrows denote large senescent cells. Scale bar = 60 μm.

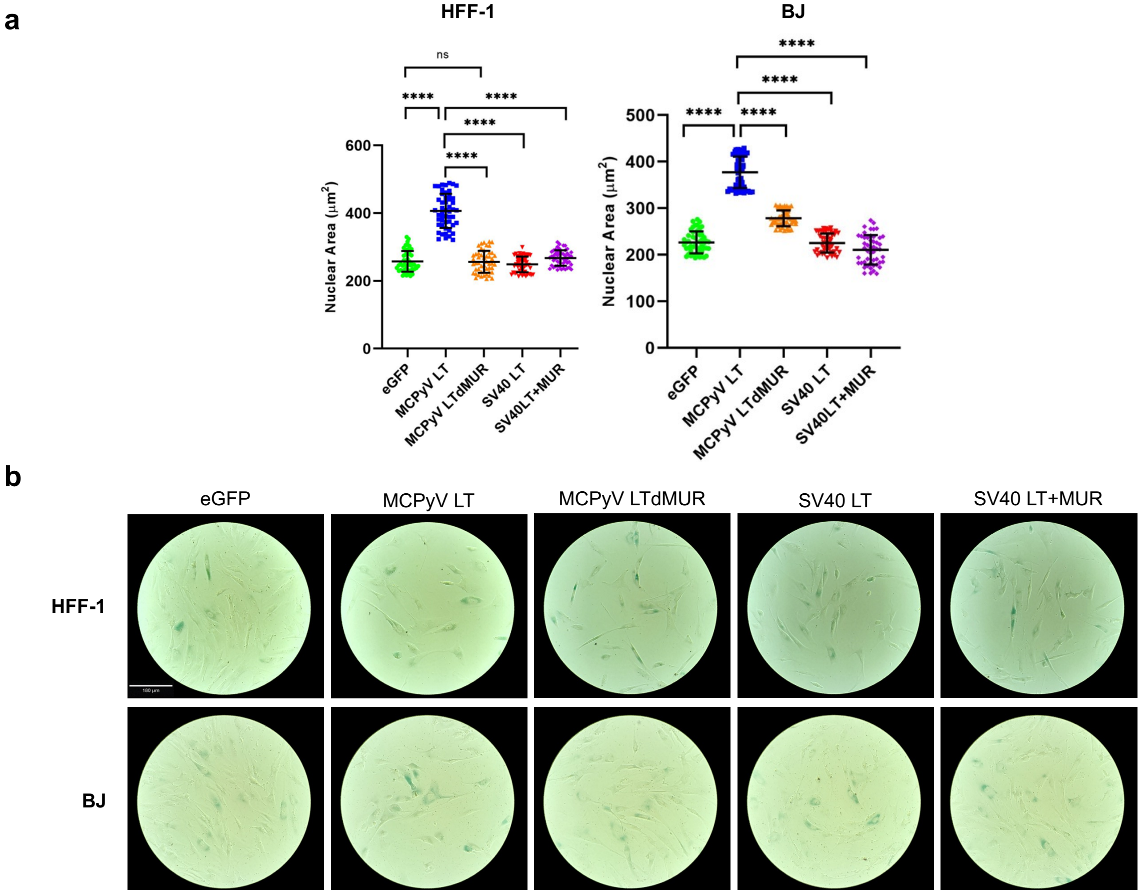

Fig. S2. MCPyV LT expression exhibits senescence-specific enlarged nucleus in HFF–1 and BJ foreskin fibroblasts. (a) Abnormal nuclear size change in MCPyV LT–expressing (GFP^+) HFF–1 (at passage 13) and BJ (at passage 9) human fibroblast cells. Nuclear size was measured 7 days after transduction of each lentiviral construct. Statistical significance was determined using the one–way ANOVA test. Standard error bars represent mean value with standard deviation, n = 50 cells. **(b)** Normal human fibroblast cell lines displayed high senescence-associated β-galactosidase (SA–β–Gal) activity. Thus, BJ–hTERT cell line was used to determine accurate gene changes in MCPyV LT–induced senescence shown in Figure 5. Scale bar = 180 μm.

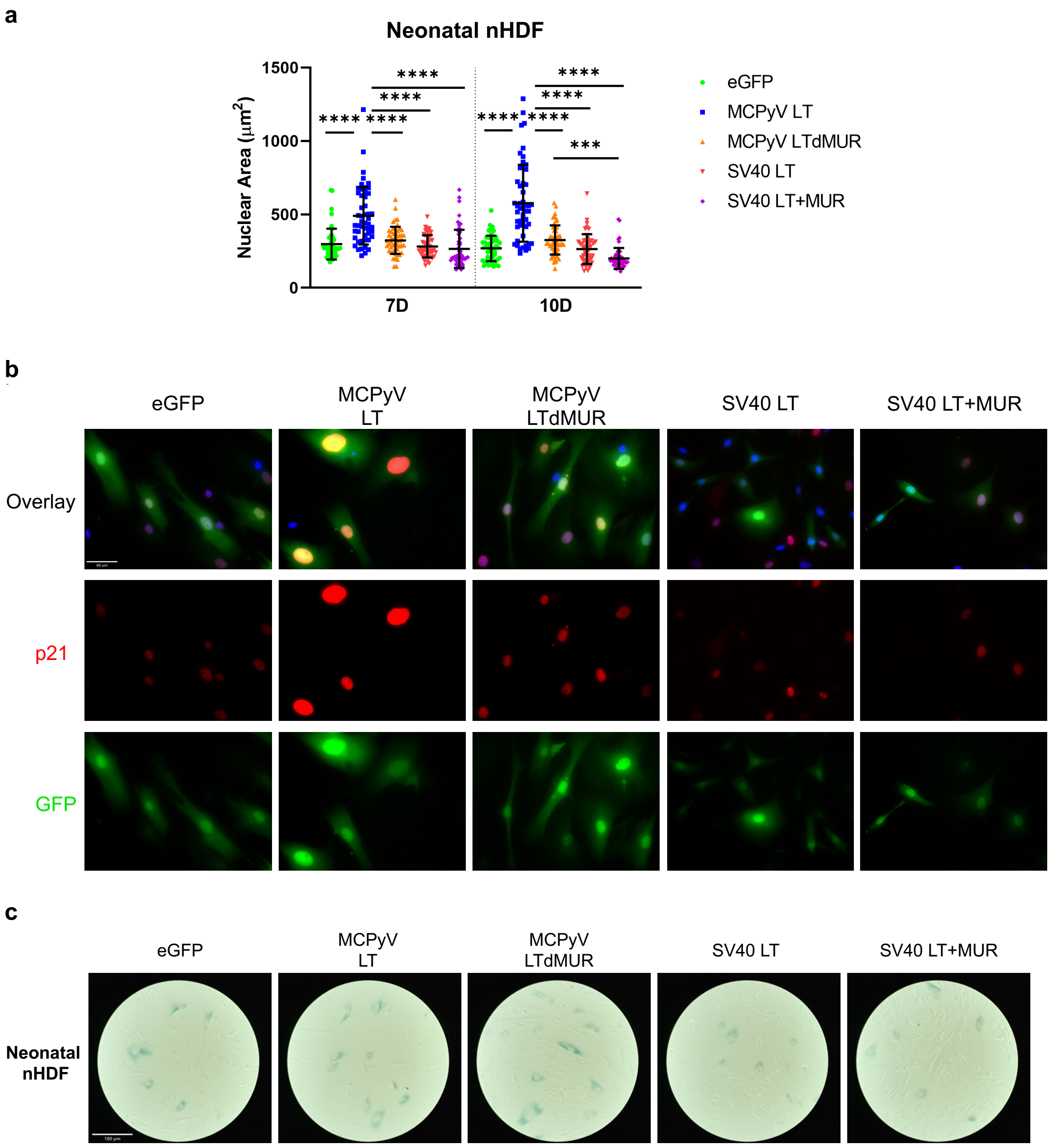

Fig. S3. MCPyV LT expression promotes senescent phenotypes in neonatal normal primary dermal fibroblast cells. (a) MCPyV LT expression induces a senescence–associated increase in nucleus size at 7 (7D) and 10 (10D) days post–transduction in neonatal normal primary dermal fibroblast (nHDF) cells. Statistical significance was determined using the one–way ANOVA test. Standard error bars represent mean value with standard deviation, n = 50 cells. **(b)** p21 is upregulated in MCPyV LT–expressing nHDF cells. Immunofluorescent analysis was conducted at 10 days post-transduction. Scale bar = 60 μm. **(c)** nHDF cells exhibit high levels of senescence–associated β–galactosidase (SA–β–Gal) background at 7 days post–transduction. Scale bar = 180 μm.

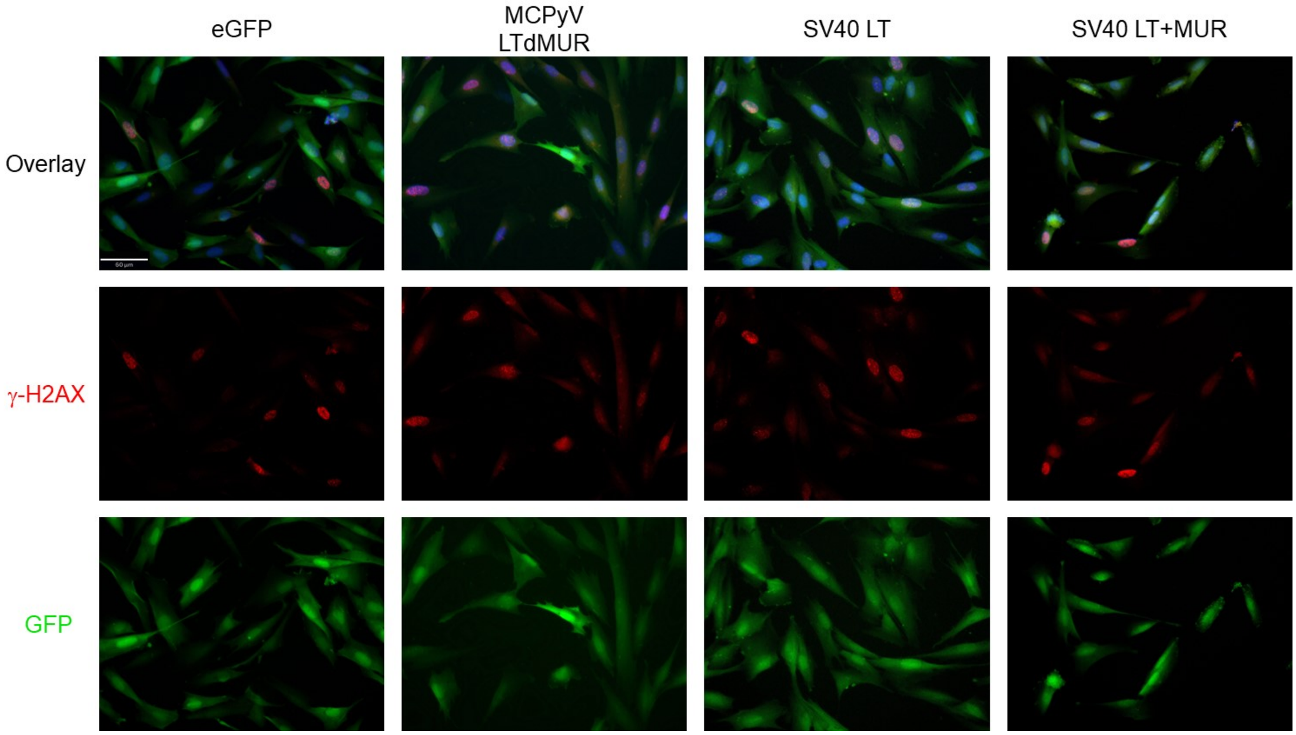

Fig. S4. Immunofluorescent analysis of γ–H2AX. Representative immunofluorescence images of nuclear γ-H2AX in eGFP, SV40LT, SV40LT+MUR and MCPyV LTdMUR–expressing cells are shown. Immunofluorescent staining of γ-H2AX (red) shows DNA damage response. Scale bar = 60 μm.

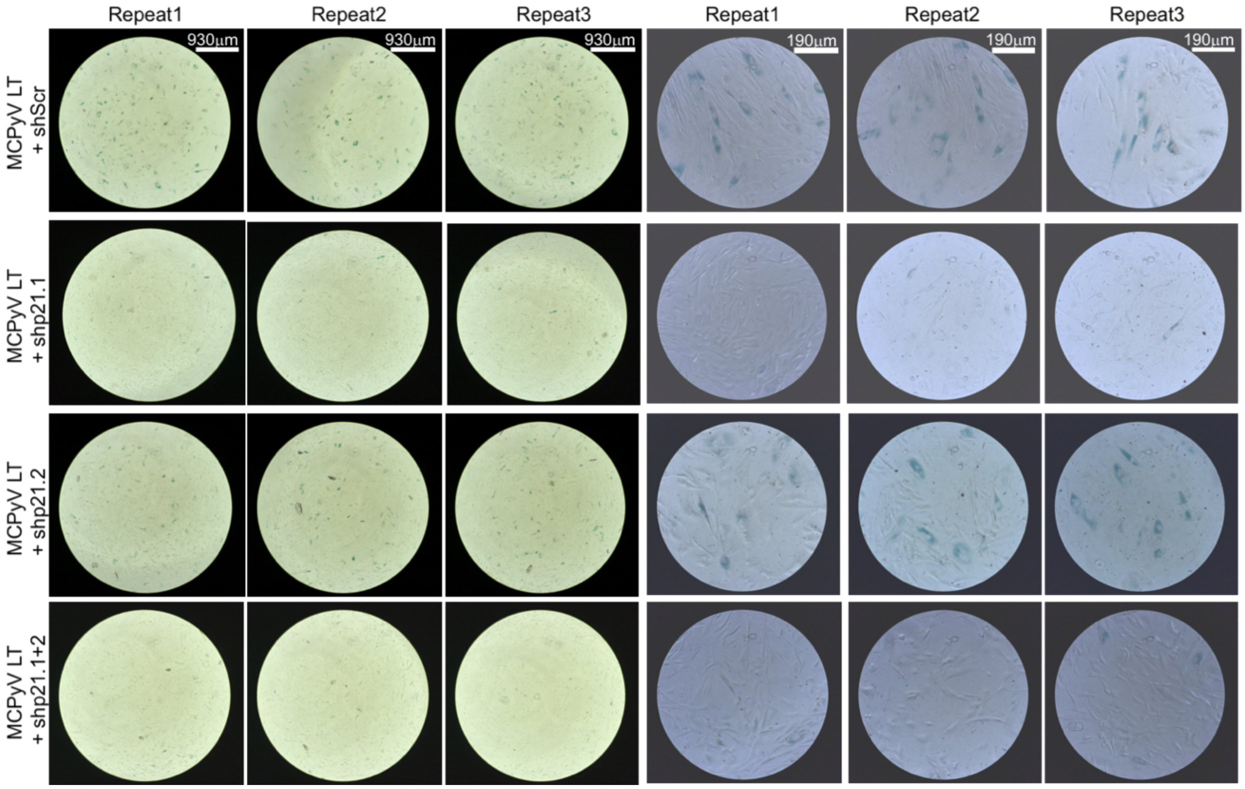

Fig. S5. MCPyV LT induces p21–dependent senescence. Knockdown of p21 was performed by short hairpin RNA (shRNA) transduction in BJ–hTERT cells expressing MCPyV LT using two shRNA lentiviral constructs, shp21.1, shp21.2. Transduction with scrambled shRNA (Scr) was used as a control. p21 knockdown largely inhibited MCPyV LT–mediated senescence.

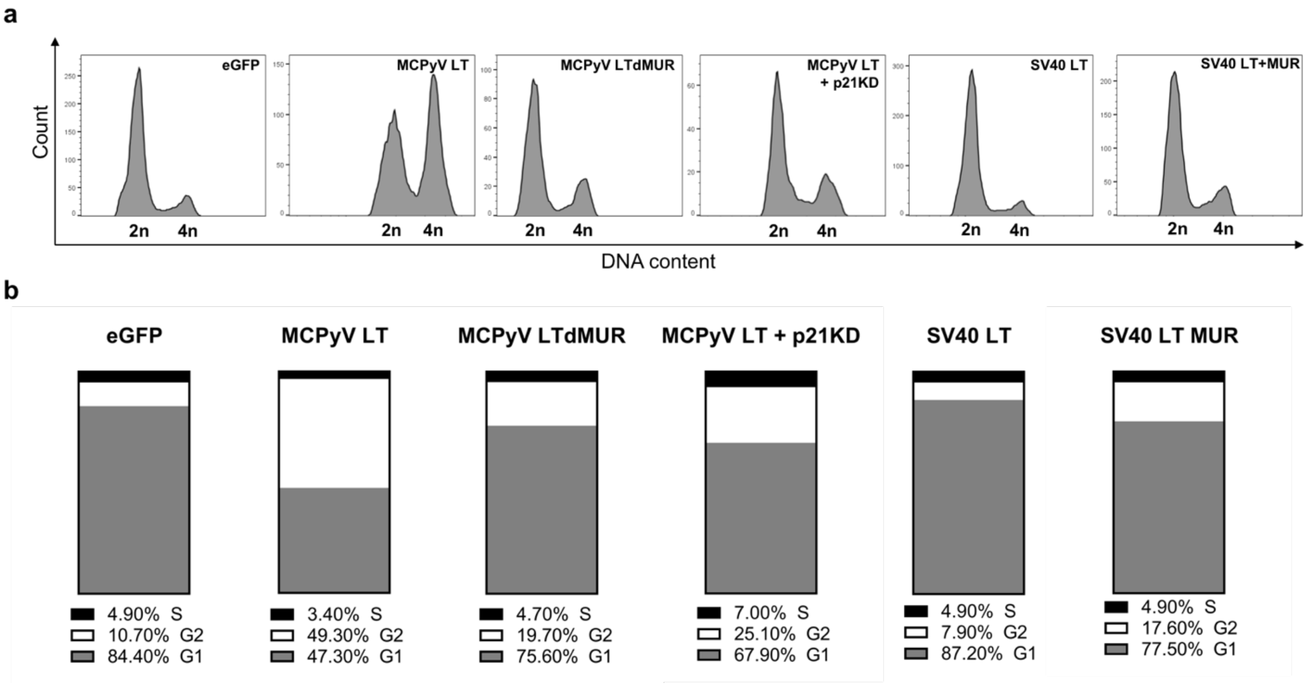

Fig. S6. MCPyV LT expression induces G2 cell cycle arrest. (a) Cell cycle analysis using flow cytometry. BJ–hTERT cells–expressing LTs were stained with Hoechst 33342 dye. Cell cycle profiles were denoted as 2n (G1) and 4n (G2). **(b)** Average percentage of cells in G1, S, and G2 phases from three independent experiments is shown. Most of MCPyV LT–expressing cells were in G2 phase. Knockdown of p21 in MCPyV LT–expressing cells allowed for cell cycle reentry.

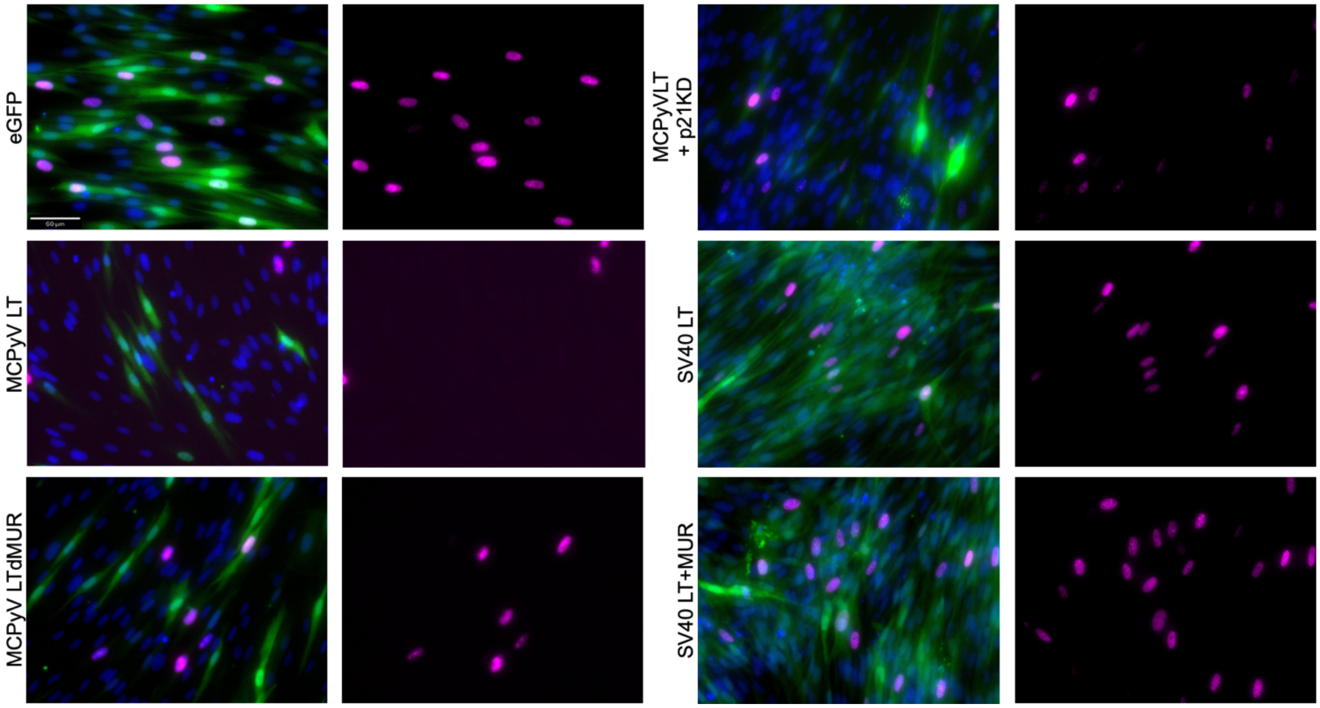

Fig. S7. MCPyV LT negatively regulates cell growth by inducing cellular senescence. Immunofluorescent imaging of EdU incorporation is shown. EdU incorporation (pink) was detected, stained with DAPI (blue), and then visualized with a REVOLVE4 fluorescence microscope. Scale bar = 60 μm.

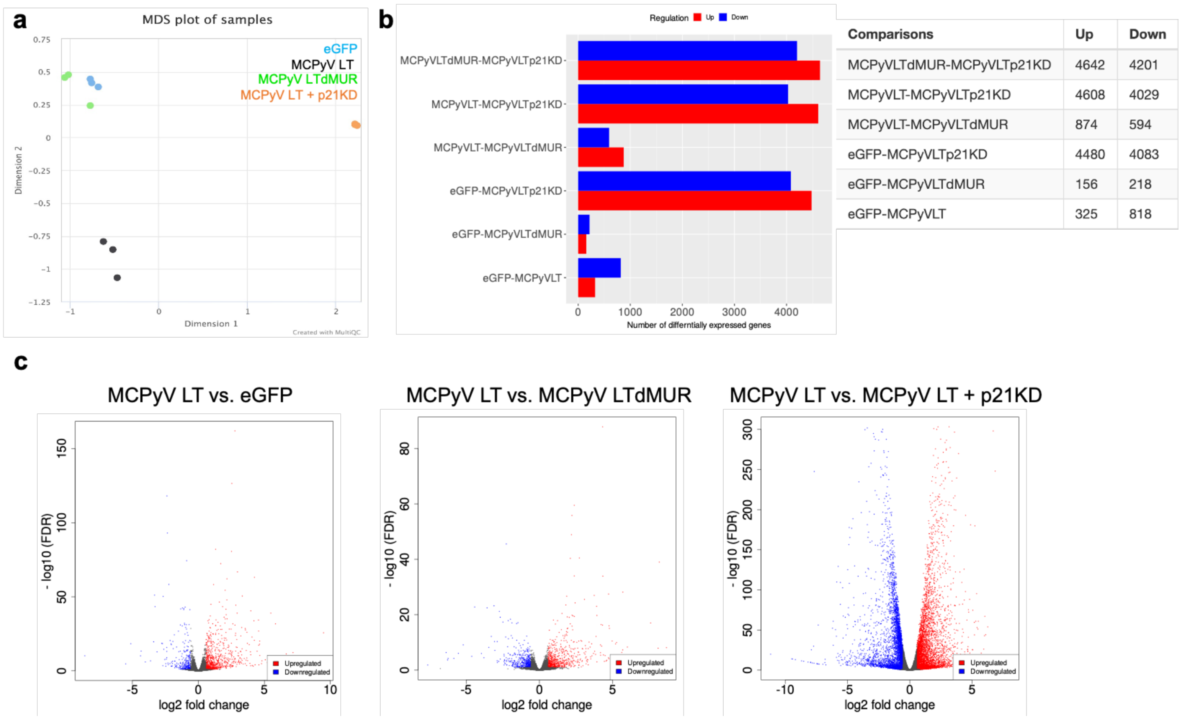

Comparisons	Up	Down
MCPyVLTdMUR-MCPyVLTp21KD	4642	4201
MCPyVLT-MCPyVLTp21KD	4608	4029
MCPyVLT-MCPyVLTdMUR	874	594
eGFP-MCPyVLTp21KD	4480	4083
eGFP-MCPyVLTdMUR	156	218
eGFP-MCPyVLT	325	818

Fig. S8: Differential gene expression analysis. (a) Multidimensional analysis plot illustrating transcriptional profiles between MCPyV LT constructs and replicates. **(b)** Differences in gene expression between samples are shown by histograms displaying the number of upregulated or downregulated genes. **(c)** Volcano plots display upregulated and downregulated differentially expressed genes (DEGs) between indicated samples. False discovery rate (FDR) cutoff was set to 0.1 and fold change cutoff was set to 1.5.

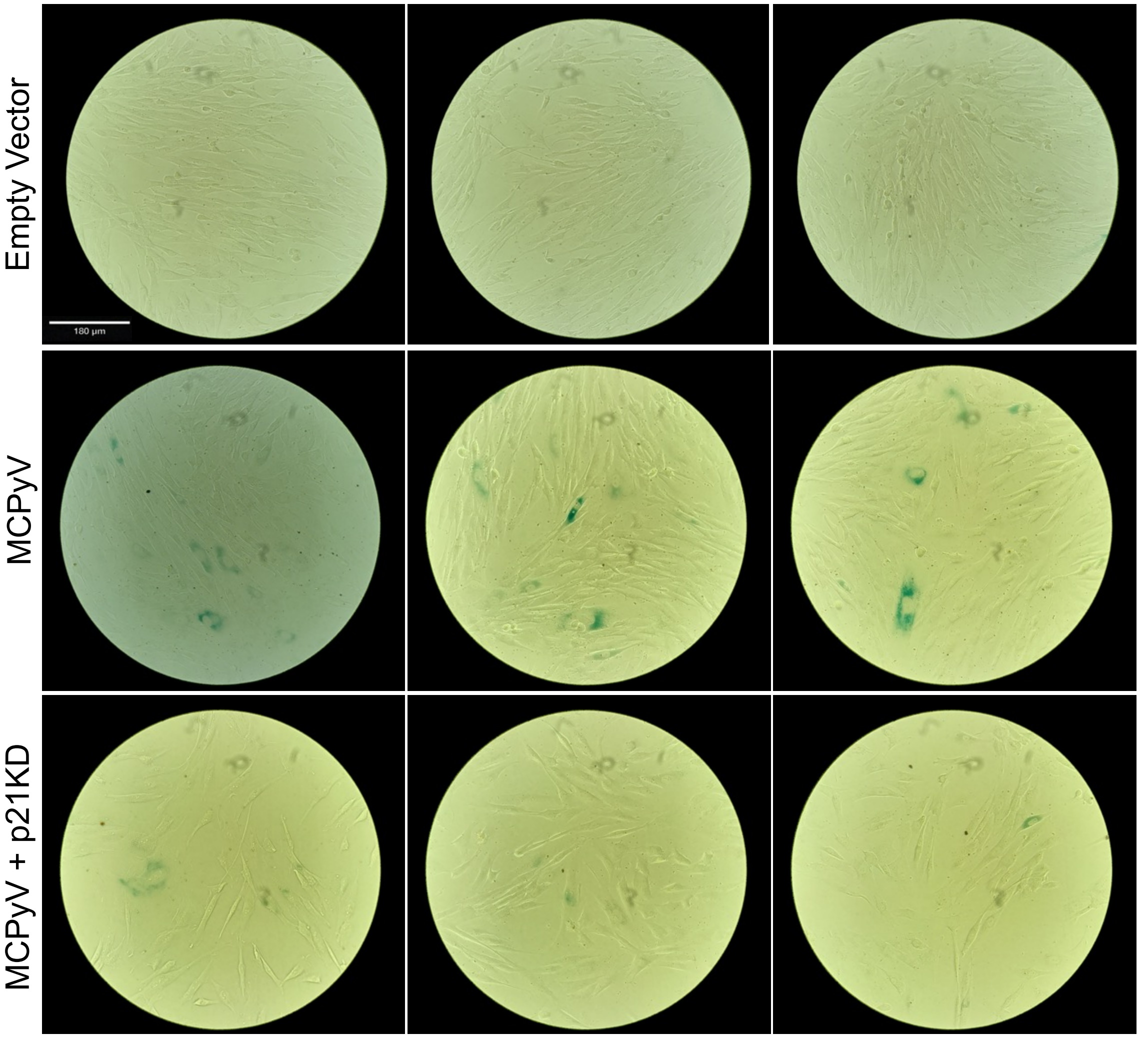

Fig. S9. MCPyV induces p21–dependent senescence. SA–β–gal staining in empty vector control or MCPyV genome transfected cells with or without p21 knockdown was visualized and photographed under phase contrast and bright field microscopy. At 14 days post transfection, p21 knockdown was performed by lentiviral shRNA transduction and SA–β–gal staining was conducted 7 days post p21 knockdown. Scale bar = 180 μm.

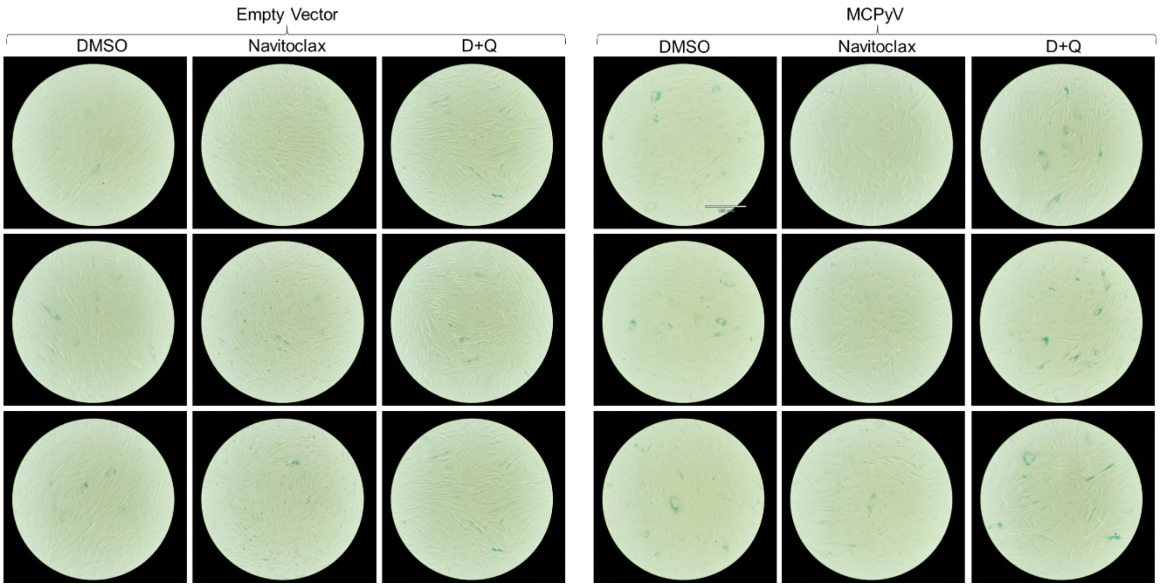

Fig. S10. Senolytic treatment inhibits MCPyV–induced cellular senescence. SA–β–gal assay. Empty vector (left) or MCPyV (right) transfected BJ-hTERT cells were grown for 14 days. Cells were then treated with DMSO, navitoclax (250 nM), or dasatinib+quercetin (D+Q, 25 nM dasatinib + 250 nM quercetin) for two days and then subjected to SA–β–gal staining. Senolytics selectively cleared senescent cells induced by MCPyV. Navitoclax treatment substantially reduced MCPyV–induced senescence. Representative images are shown. Scale bar = 180 μm.

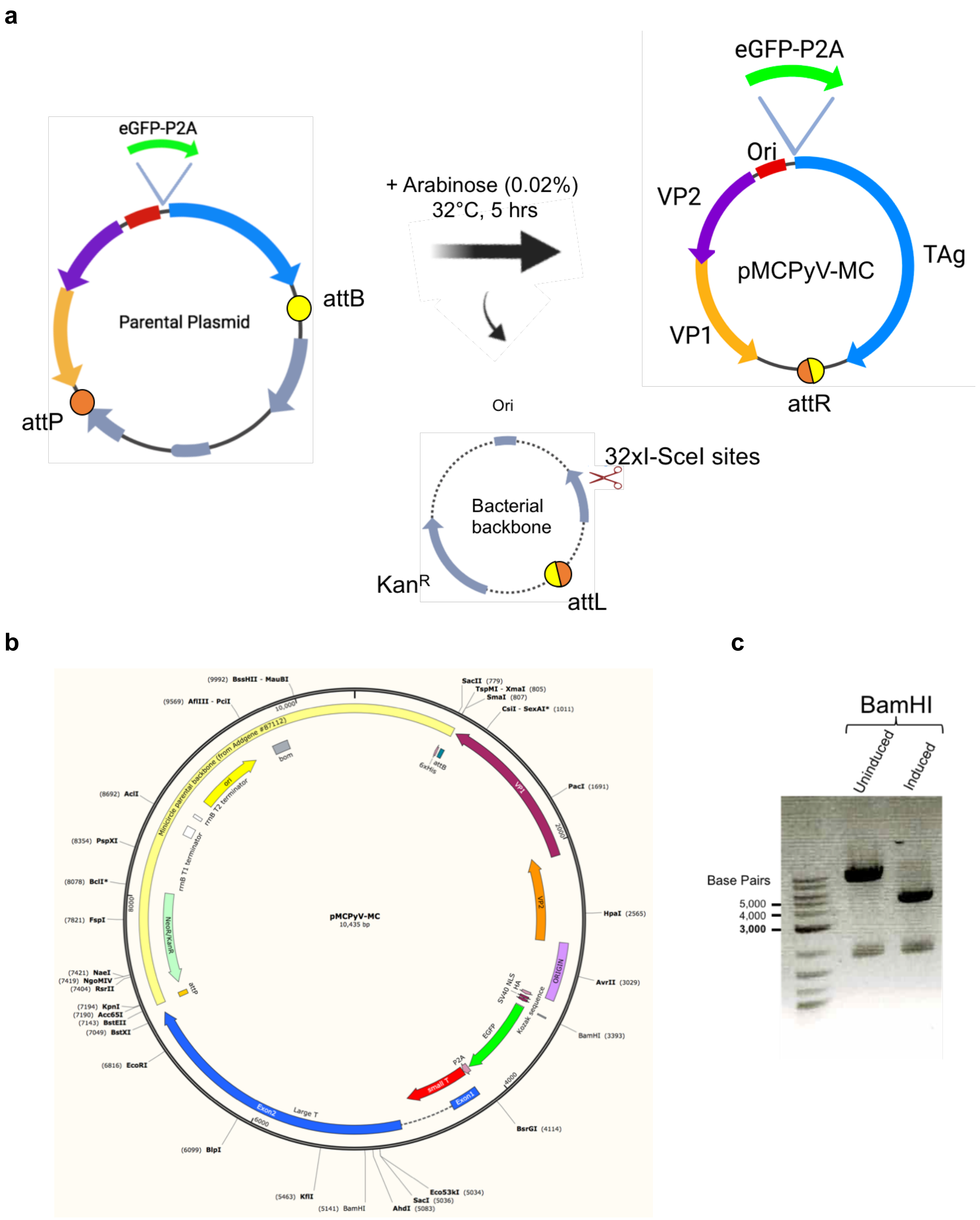

Fig. S11. MCPyV minicircle genome.
(a) Summarized representation of the strategies used for minicircle purification. **(b)** A construct of MCPyV minicircle genome. **(c)** Electrophoresis of MCPyV minicircle clone. MCPyV genome was isolated before and after induction by adding 0.02% arabinose and digested with BamHI.

Table S1. Primers used in this study.

Name	Sequence (5' to 3')	Notes
HFori_AvrII.F	CAACTTGGCTGCCTAGGTG	eGFP-MCPyV-HFwt construct
Ori_HA_NLS_GFP11.F	GCATATAGACAAGACCATGTATCCCTATGACGTG	eGFP-MCPyV-HFwt construct
Ori_HA_NLS_GFP11.R	CACGTCATAGGGATACATGGTCTTGTCTATATGC	eGFP-MCPyV-HFwt construct
P2A_MCVER.F	GAGAACCCTGGACCTGATTTAGTCCTAAATAG	eGFP-MCPyV-HFwt construct
P2A_MCVER.R	CTATTTAGGACTAAATCAGGTCCAGGGTTCTC	eGFP-MCPyV-HFwt construct
MCV350.1605-1585	CAGCATGGCTAAGATAATCAG	eGFP-MCPyV-HFwt construct
MCVHFmcF-SmaI	CCCCGGGCGCGGGAATGCATGAAATAATTCTCATAATTCTTGTGTTTGGC	pMCPyV-MC construct
MCVHFmcR-BstEII	CTCAAAGGTTACCCCAGTTGGGGCGGGCCCTATTCAGTTAAGTAGGCCCCAG	pMCPyV-MC construct
Exon1.R	TCCAAAGGGTGTTCAATTCC	pMCPyV-MC construct
eGFPqPCR.F	GCAAGGGCGAGGAGCTGTTCAC	pMCPyV-MC construct
CDKN1A p21.F	GACACCACTGGAGGGTGACT	RT-qPCR
CDKN1A p21.R	CAGGTCCACATGGTCTTCCT	RT-qPCR
CXCL1.F	GAAAGCTTGCCTCAATCCTG	RT-qPCR
CXCL1.R	CACCAGTGAGCTTCCTCCTC	RT-qPCR
CXCL2.F	AACTGCGCTGCCAGTGCT	RT-qPCR
CXCL2.R	CCCATTCTTGAGTGTGGCTA	RT-qPCR
IL6.F	CCGGGAACGAAAGAGAAGCT	RT-qPCR
IL6.R	GCGCTTGTGGAGAAGGAGTT	RT-qPCR
shP21.1-F	CCGGCGCTCTACATCTTCTGCCTTACTCGAGTAAGGCAGAAGATGTAGAGCGTTTTTG	shRNA (TRCN0000287021)
shP21.1-R	AATTCAAAAACGCTCTACATCTTCTGCCTTACTCGAGTAAGGCAGAAGATGTAGAGCG	shRNA (TRCN0000287021)
shP21.2-F	CCGGGACAGATTTCTACCACTCCAACTCGAGTTGGAGTGGTAGAAATCTGTCTTTTTG	shRNA (TRCN0000040126)
shP21.2-R	AATTCAAAAAGACAGATTTCTACCACTCCAACTCGAGTTGGAGTGGTAGAAATCTGTC	shRNA (TRCN0000040126)
GAPDH.F	CCTCCCGCTTCGCTCTCT	RT-qPCR
GAPDH.R	CTGGCGACGCAAAAGAAGA	RT-qPCR
RNase P.F	GCGGAGGGAAGCTCATCAG	RT-qPCR, MCPyV qPCR
RNase P.R	CTGGCCCTAGTCTCAGACCTT	RT-qPCR, MCPyV qPCR
MCV350.1605-1585	CAGCATGGCTAAGATAATCAG	MCPyV qPCR
MCV350.1505-1528	CAGCAAGTTTTACAAGCACTCCACC	MCPyV qPCR
eGFPqPCR.F	GCAAGGGCGAGGAGCTGTTCAC	MCPyV qPCR
eGFPqPCR.R	GGTGGCATCGCCCTCGCCCTC	MCPyV qPCR
HFligRep.F1	GCTGCTGCAGAGTTCCTCCTATATG	MCPyV qPCR
HFligRep.R	GGCTGCAGATACAATCAAACCTAG	MCPyV qPCR
pLKOmChe_BHI.F	CAGGGGGATCCATGGTGAGCAAGGGCGAGG	pLKO.1 mCherry-Puro construct
pLKO_KpnI_R	GGTCTTAAAGGTACCGATGCATGGGGTC	pLKO.1 mCherry-Puro construct
P2Apuro.F	GAACCCTGGACCTAGCGCTATGACCGAGTACAAGCCC	pLKO.1 mCherry-Puro construct
mCherryP2A.F	GACGAGCTGTACAAGGGAAGCGGAGCTAC	pLKO.1 mCherry-Puro construct
mCherryP2A.R	GTAGCTCCGCTTCCCTTGTACAGCTCGTC	pLKO.1 mCherry-Puro construct

Supplementary Dataset S1(separate file). The comprehensive functional enrichment analysis.
